# The aryl hydrocarbon receptor promotes differentiation during mouse preimplantational embryo development

**DOI:** 10.1016/j.stemcr.2021.08.002

**Published:** 2021-09-02

**Authors:** Ana Nacarino-Palma, Francisco J. González-Rico, Claudia M. Rejano-Gordillo, Ana Ordiales-Talavero, Jaime M. Merino, Pedro M. Fernández-Salguero

**Affiliations:** 1 Departamento de Bioquímica y Biología Molecular, Facultad de Ciencias, Universidad de Extremadura, Avenida de Elvas s/n, 06071 Badajoz, Spain; 2 Instituto de Investigación Biosanitaria de Extremadura (INUBE), Avenida de la Investigación s/n, 06071 Badajoz, Spain

**Keywords:** aryl hydrocarbon receptor, embryo differentiation, pluripotency, Hippo, preimplantation

## Abstract

Mammalian embryogenesis is a complex process controlled by transcription factors that regulate the balance between pluripotency and differentiation. Transcription factor aryl hydrocarbon receptor (AhR) regulates OCT4/POU5F1 and NANOG, both essential controllers of pluripotency, stemness and early embryo development. Molecular mechanisms controlling OCT4/POU5F1 and NANOG during embryogenesis remain unidentified. We show that AhR regulates pluripotency factors and maintains the metabolic activity required for proper embryo differentiation. AhR-lacking embryos (*AhR*^−/−^) showed a pluripotent phenotype characterized by a delayed expression of trophectoderm differentiation markers. Accordingly, central pluripotency factors OCT4/POU5F1 and NANOG were overexpressed in *AhR*^−/−^ embryos at initial developmental stages. An altered intracellular localization of these factors was observed in the absence of AhR and, importantly, *Oct4* had an opposite expression pattern with respect to AhR from the two-cell stage to blastocyst, suggesting a negative regulation of OCT4/POU5F by AhR. We propose that AhR is a regulator of pluripotency and differentiation in early mouse embryogenesis.

## Highlights

• AhR regulates pluripotency factors OCT4 and NANOG during early embryo differentiation
• AhR lacking embryos (*AhR^−/−^*) show a pluripotent phenotype
• Pluripotent phenotype of *AhR^−/−^* embryos show enhanced glycolytic metabolism

## Teaser

In this article, Fernández-Salguero and colleagues show that transcription factor AhR regulates pluripotency factors and maintains the metabolic activity required for proper embryo differentiation. AhR-lacking embryos (*AhR*^−/−^) showed a pluripotent phenotype characterized by a delayed expression of trophectoderm differentiation markers. Accordingly, central pluripotency factors were overexpressed in *AhR*^−/−^ embryos at initial developmental stages.

## Introduction

The aryl hydrocarbon receptor (AhR) is a transcription factor with important toxicological and physiological implications and with recently discovered roles in pluripotency and stemness (Ko and Puga, 2017; Mulero-Navarro and Fernandez-Salguero, 2016; Roman et al., 2018). Several studies support this receptor as an important regulator of the balance between pluripotency and differentiation under physiological conditions and in tumor cells. Indeed, AhR activation by the carcinogen TCDD (2,3,7,8-tetraclorodibenzo-p-dioxin) during mouse pregnancy blocked the ability of hematopoietic stem cells for long-term self-renewal (Laiosa et al., 2015). Similarly, sustained AhR activation during early differentiation of mouse embryonic stem cells impaired signaling critical for the ontogeny of cardiac mesoderm and cardiomyocyte functioning (Wang et al., 2016). Previous work from our laboratory using human NTERA-2 cells (pluripotent human testicular embryonal carcinoma cell line; ATCC: CRL-1973) revealed that AhR supports cell differentiation through the transcriptional repression of retrotransposable *Alu* elements located in the promoter region of pluripotency genes *OCT4* and *NANOG* (Morales-Hernandez et al., 2016). On the contrary, receptor deficiency in mice produces a more undifferentiated phenotype that enhances the regenerative potential of the lung (Morales-Hernandez et al., 2017) and the liver (Moreno-Marin et al., 2017) upon acute damage.

A distinguishing feature of preimplantational development is the gradual loss of totipotency suffered by embryo blastomeres. Throughout embryonic development from zygote to blastocyst, stem cells will restrict their fate through cellular differentiation after successive rounds of cell division. Three different cell lineages actually exist in the mature blastocyst; namely, trophectoderm, epiblast, and primitive endoderm (Chazaud and Yamanaka, 2016). In the first cell fate decision, asymmetric divisions in the initial embryo generate cells that differ in their cellular properties, location within the embryo, and phenotypic outcome (Fleming, 1987). Subcellular heterogeneities in tensile forces, caused by actomyosin cortical networks, drive apical constriction. These contractile forces in the embryo are crucial in determining both the position and first spatial segregation of cells (Maître, 2017; Samarage et al., 2015). Surface cells will differentiate into the trophectoderm (TE) to originate the precursor lineage of the placenta, whereas internal cells will form the inner cell mass (ICM) that will differentiate in the second cell division to form the primitive endoderm (PE). Several signaling networks are responsible for coordinating the myriad events needed to control the balance between differentiation and pluripotency in embryogenesis. Transcription factors OCT4/POU5F1 (hereafter OCT4), SOX2, and NANOG constitute the “central pluripotency network” (Maître, 2017; Samarage et al., 2015). These pluripotency factors are initially expressed in all cells of the morulae, with their expression becoming gradually restricted to the ICM after the first cell fate decision (Boyer et al., 2005). Establishment of TE fate program in outside cells is regulated by the Hippo pathway, which acts as a sensor of cell polarity.

Outside cells have asymmetric cell-cell contacts that accumulate apical polarity proteins inhibiting the activity of the tight junction proteins AMOT (Angiomotin) and the Hippo pathway kinases Large tumor suppressor kinases 1/2 (LATS1/2) (Bedzhov et al., 2014; Boyer et al., 2006). As a result, hypophosphorylated Yes-activated protein (YAP) is translocated to the nucleus and the TE cell fate program is activated with an increase in CDX2 expression through TEAD4. In inside cells, symmetric cell-cell contacts prevent the establishment of an apical domain. AMOT proteins are then activated and distributed along the general membrane in adherent junctions in NF2/α-catenin/β-catenin/E-cadherin complexes. In addition, LATS1/2 become activated, the resulting phoshor-YAP (pYAP) is excluded from the nucleus, and OCT4, once expressed, initiates the pluripotency program determining the ICM fate (Leung and Zernicka-Goetz, 2013).

Knowing how cell fate is specified in the preimplantation embryo may help us to understand the mechanisms that regulate pluripotency and differentiation of stem cells of embryonic origin as well as those arising from tumors. Interestingly, early and previous reports have shown that AhR-null mice have a reduced fertility, producing fewer numbers of born-alive pups as compared with AhR-expressing littermates. In fact, such a phenotype seems to be at least partially due to an increase in embryo resorption and an impaired ability to complete the preimplantation program up to the blastocyst stage (Manzanares and Rodriguez, 2013; Paramasivam et al., 2011).

Here, we have studied how AhR affects the early stages of preimplantation during mouse development in an attempt to further understand receptor functions in pluripotency and differentiation. We have found that AhR has pro-differentiation activities in the early mouse embryo that are needed to specify the different cell fates from the one-cell to the blastocyst stage. Our results suggest that AhR has relevant roles in embryonic stem cell differentiation through the control of genes responsible for maintaining a pluripotent status and by a adjusting a proper balance between glycolytic and mitochondrial metabolisms. AhR deficiency may thus negatively affect embryo progression during preimplantation, eventually compromising normal development and viability.

## Results

### AhR expression and localization is modulated throughout embryonic development

**Figure 1:**
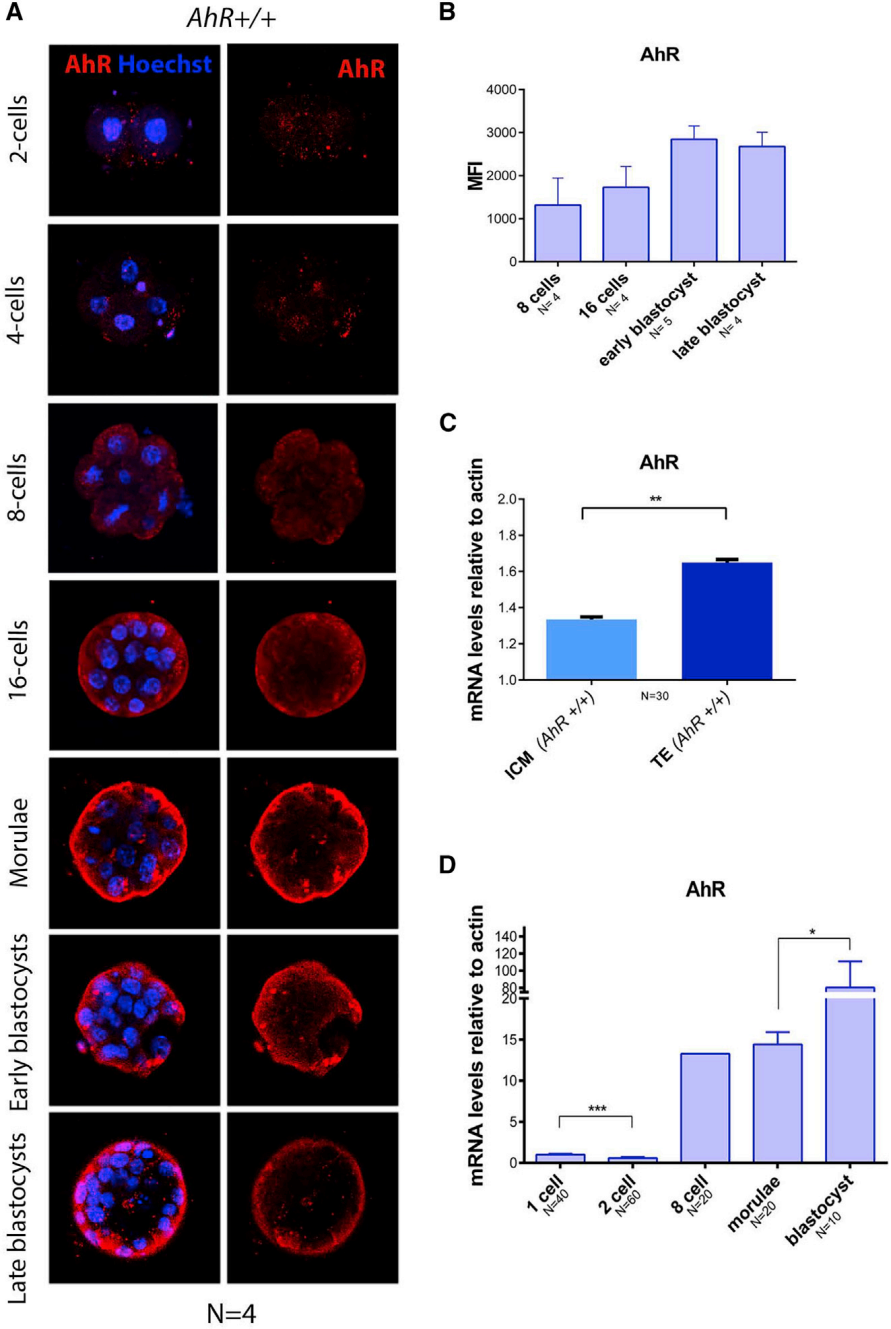
AhR expression increases during embryo differentiation (A) Immunofluorescence analysis of AHR at the indicated embryonic stages. Whole *AhR*^+/+^ embryos (n = 4) were stained using a specific AHR antibody. Hoechst staining was used to label cell nuclei. Confocal microscopy was used for detection. Scale bar, 20 μm. (B) Immunofluorescence was quantified by calculating the mean fluorescence intensity (MFI) for each developmental stage (the replicates are indicated in the x axis). (C) *AhR* mRNA expression was quantified by qRT-PCR using RNA purified from TE or ICM fractions previously separated by MACS. (D) *AhR* mRNA expression was quantified by qRT-PCR in *AhR*^+/+^ embryos at the indicated developmental stages using total RNA and the specific primers indicated in Table S1. qRT-PCR was normalized by the expression of *β*-*actin* and is represented as 2^−ΔΔCt^. ^∗^p < 0.05; ^∗∗^p < 0.01; ^∗∗∗^p < 0.001. Data are shown as mean ± SD. The experiments were performed at least four times, and number of embryos analyzed is indicated on the x axis.

To analyze the role of AhR in early embryo differentiation, we first analyzed AHR expression levels along different embryonic stages. Confocal immunofluorescence analysis showed that AHR was significantly and steadily expressed as differentiation progressed from two-cell zygote to blastocyst (Figures 1A and 1B). Regarding AHR localization within the embryo, the immunofluorescence analysis revealed a generalized expression in all cells up to the morulae stage with some cells having nuclear AHR. However, as differentiation progressed to the early and late blastocyst, AHR was only detected in the external blastomeres, being almost absent in those cells forming the ICM (Figure 1A). To further support this finding, we separated inner and outer (TE) blastomeres from blastocysts using magnetic-activated cell sorting and analyzed AhR expression in both fractions. The results confirmed that *AhR* mRNA levels were significantly higher in TE blastomeres than in ICM blastomeres (Figure 1C). Consistently, *AhR* mRNA expression significantly increased during differentiation from zygote to blastocyst at the transcriptional level (Figure 1D). AHR expression, as detected by immunofluorescence, was specific considering the complete lack of signal in AhR-null embryos of similar developmental stages (Figure S1). These results indicated that the expression of the AhR is modulated throughout early embryonic development and that its embryonic localization changes with differentiation.

### AhR deficiency induces upregulation of pluripotency genes during early embryo development

**Figure 2:**
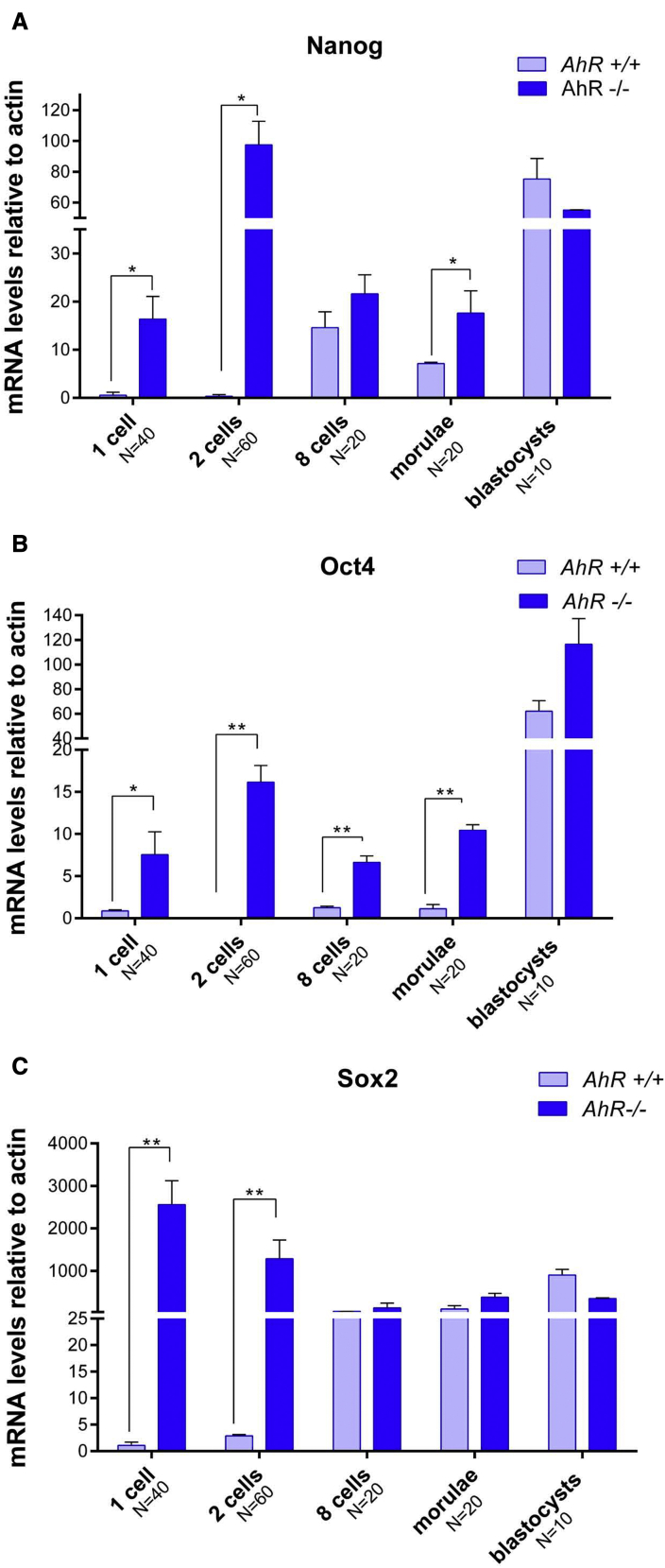
Pluripotency factors are upregulated in AhR-null embryos (A–C) *AhR*^+/+^ and *AhR*^−/−^ embryos were obtained at the indicated embryonic stages and used to quantify the mRNA expression of *Nanog* (A), *Oct4* (B), *and Sox2* (C) by qRT-PCR. Expression levels were normalized by *β*-*actin* and are represented as 2^−ΔΔCt^. ^∗^p < 0.05; ^∗∗^p < 0.01. Data are shown as mean ± SD. The experiments were performed at least four times, and number of embryos analyzed is indicated on the x axis.

To assess whether AhR participates in the maintenance of pluripotency during the early stages of embryogenesis, we next analyzed the levels of pluripotency factors throughout preimplantation in wild-type and AhR-null embryos. *AhR*^−/−^ embryos showed significantly higher *Nanog* and *Oct4* mRNA levels compared with *AhR*^+/+^ embryos from one-cell zygote until the morulae stage (Figures 2A and 2B). In blastocysts, *Oct4* mRNA expression kept rising in AhR-null embryos while *Nanog* mRNA levels became balanced among both genotypes (Figures 2A and 2B). Regarding *Sox2*, embryos lacking AhR also had higher mRNA expression of this pluripotency factor at the beginning of embryogenesis (e.g., one-cell and two-cell stages) to decrease to similar levels in both genotypes from eight-cell to blastocyst (Figure 2C). Thus, AhR plays a role in controlling the expression of genes known to regulate pluripotency and differentiation during embryo development.

### AhR modulates the localization of pluripotency factors during embryogenesis

**Figure 3:**
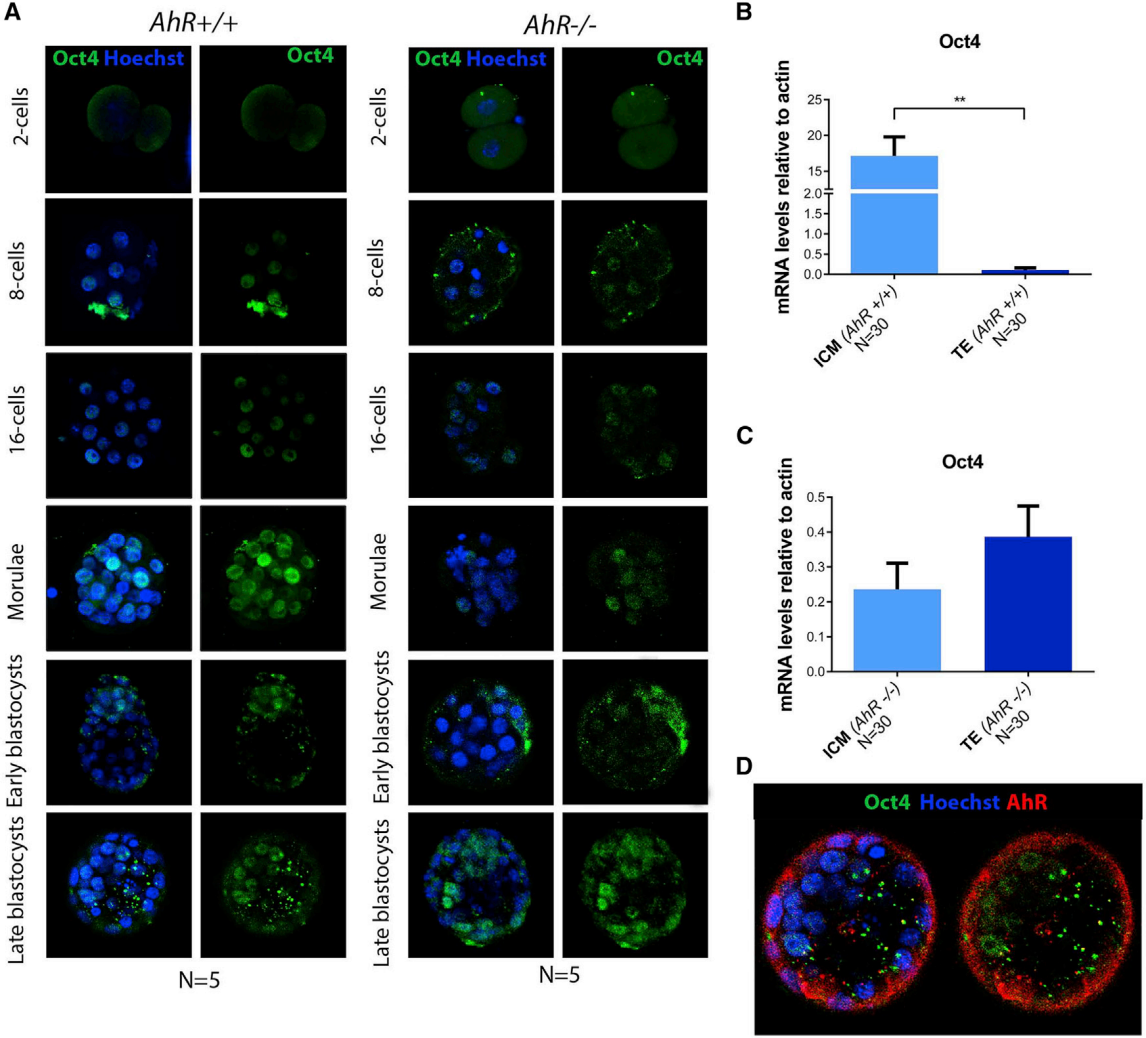
AhR depletion alters OCT4 cellular distribution through embryogenesis (A) Immunofluorescence analysis of OCT4 at the indicated embryonic stages. Whole embryos were stained using a specific antibody. Hoechst was used to stain cell nuclei. Scale bar, 20 μm. (B and C) *Oct4* mRNA expression was quantified by qRT-PCR using mRNA purified from TE and ICM fractions separated by MACS using the specific primers indicated in Table S1. mRNA expression was normalized by *β-actin* and is represented as 2^−ΔΔCt.^. (D) Immunofluorescence analysis of OCT4 (green) and AHR (red) in embryos at the blastocyst stage. Scale bar, 10 μm. ^∗∗^p < 0.01. Data are shown as mean ± SD. Confocal microscopy was used for detection. The experiments were performed at least three times, and number of embryos analyzed is indicated on the x axis.

To investigate how AhR affects protein levels and localization of pluripotency factors, we carried out immunofluorescence analysis for OCT4 and NANOG in wild-type and AhR-null embryos during early development. The results showed changes in localization of OCT4 and NANOG upon the presence of the AhR. In wild-type embryos, OCT4 had nuclear localization from the two-cell stage up to the blastocyst stage, when it mainly localized in ICM cells (Figure 3A). Embryos lacking AhR showed nuclear localization of OCT4 throughout most stages of development and up to late blastocyst (Figure 3A). We then decided to analyze OCT4 expression at the mRNA level in isolated blastomeres from the ICM and TE (Figures 3B and 3C). We found that in *AhR*^−/−^ embryos, there were no significant differences in OCT4 mRNA expression between ICM and TE blastomeres (Figure 3C), as was observed in *AhR*^+/+^ embryos (Figure 3B). These results indicate that AhR may be needed to regulate the location of OCT4 within the embryo, which could affect differentiation and cell fate. The fact that OCT4 has a location pattern opposite to that of AhR (Figures 1 and 3D) suggests that AhR may exert a negative regulation on OCT4 to drive embryo differentiation.

**Figure 4:**
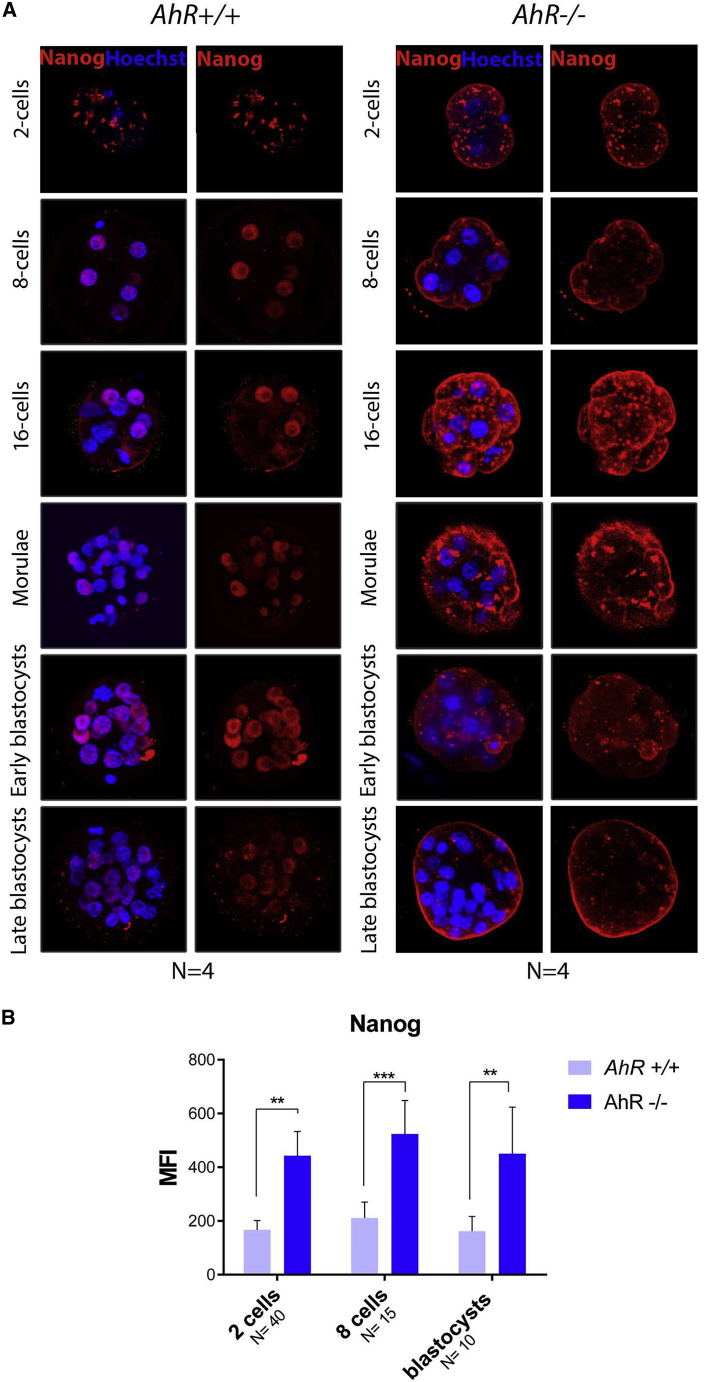
NANOG distribution in the embryo is altered in the absence of AhR (A) Immunofluorescence analysis of NANOG at the indicated embryonic stages. Whole embryos were stained using a specific antibody. Hoechst was used for staining cell nuclei. Scale bars, 20 μm. (B) Immunofluorescence was quantified by calculating the mean fluorescence intensity (MFI). ^∗∗^p < 0.01; ^∗∗∗^p < 0.001. Data are shown as mean ± SD. The experiments were performed at least four times, and number of embryos analyzed is indicated on the x axis.

NANOG localization in *AhR*^+/+^ embryos was also modified in the absence of AhR. The dotted and regular pattern showed by this protein from zygote to four-cell stage in AhR wild-type embryos remained in AhR-null embryos until the 16-cell stage (Figure 4). While in AhR wild-type embryos a polarized and nuclear distribution of NANOG was observed between ICM and TE from morulae on, a delocalization of this pluripotency factor was evident in AhR-lacking embryos (Figure 4). Quantification of immunofluorescence signals revealed that global NANOG expression was significantly higher in *AhR*^−/−^ than in *AhR*^+/+^ embryos (Figure 4B). These data suggest that, in addition to OCT4, AhR could also regulate NANOG expression during embryo development.

### AhR-null embryos show Hippo signaling upregulation

**Figure 5:**
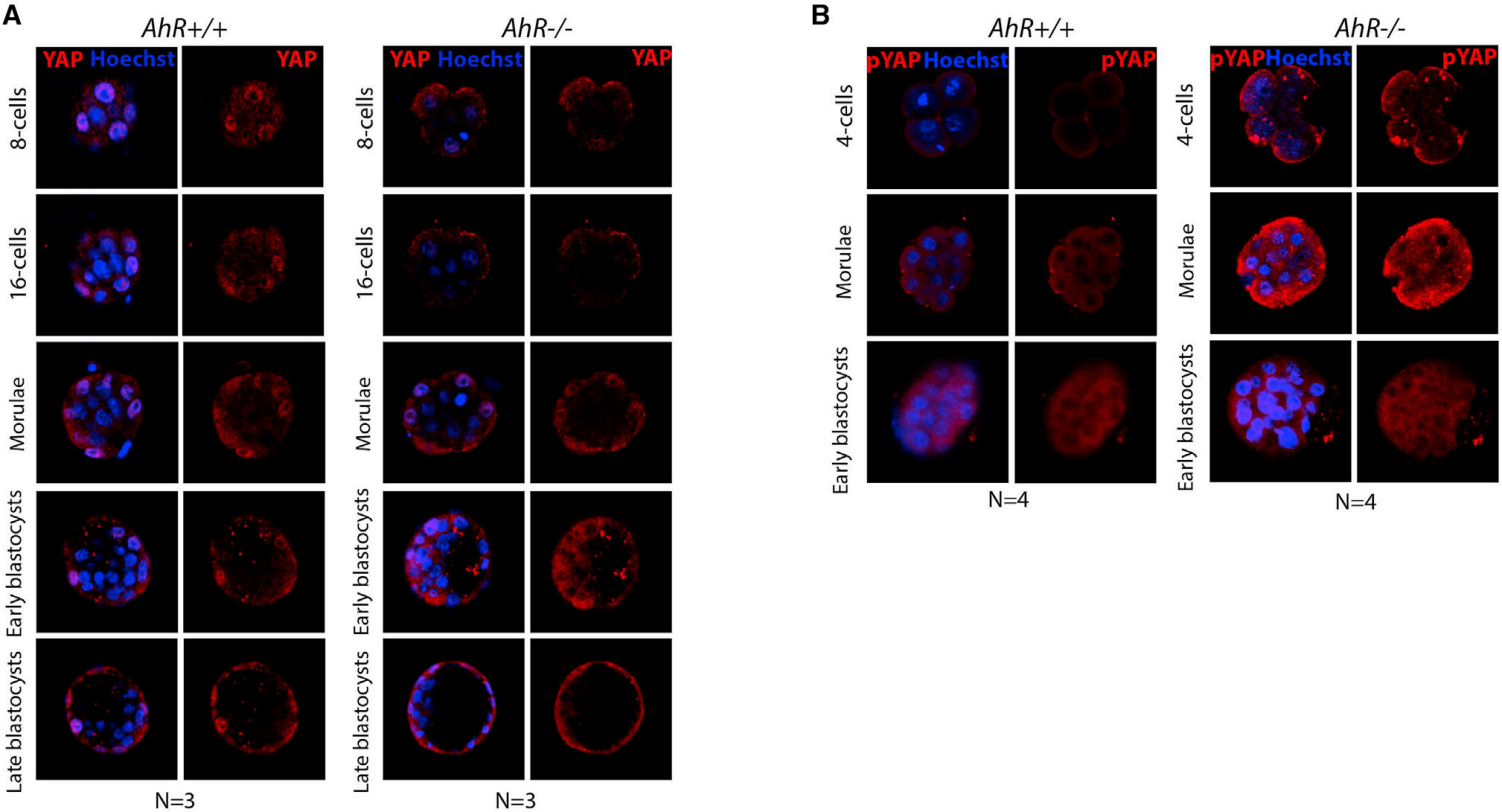
AhR deficiency alters localization of the Hippo effector YAP (A and B) Immunofluorescence analysis of YAP (A) and phosphor-YAP (pYAP) (B) at the indicated developmental stages in *AhR*^+/+^ and *AhR*^−/−^ embryos. Whole embryos were stained using specific antibodies for YAP or pYAP. Hoechst was used for staining cell nuclei. Confocal microscopy was used for detection. The experiments were performed at least three (A) and four (B) times. Scale bars, 20 μm.

**Figure 6:**
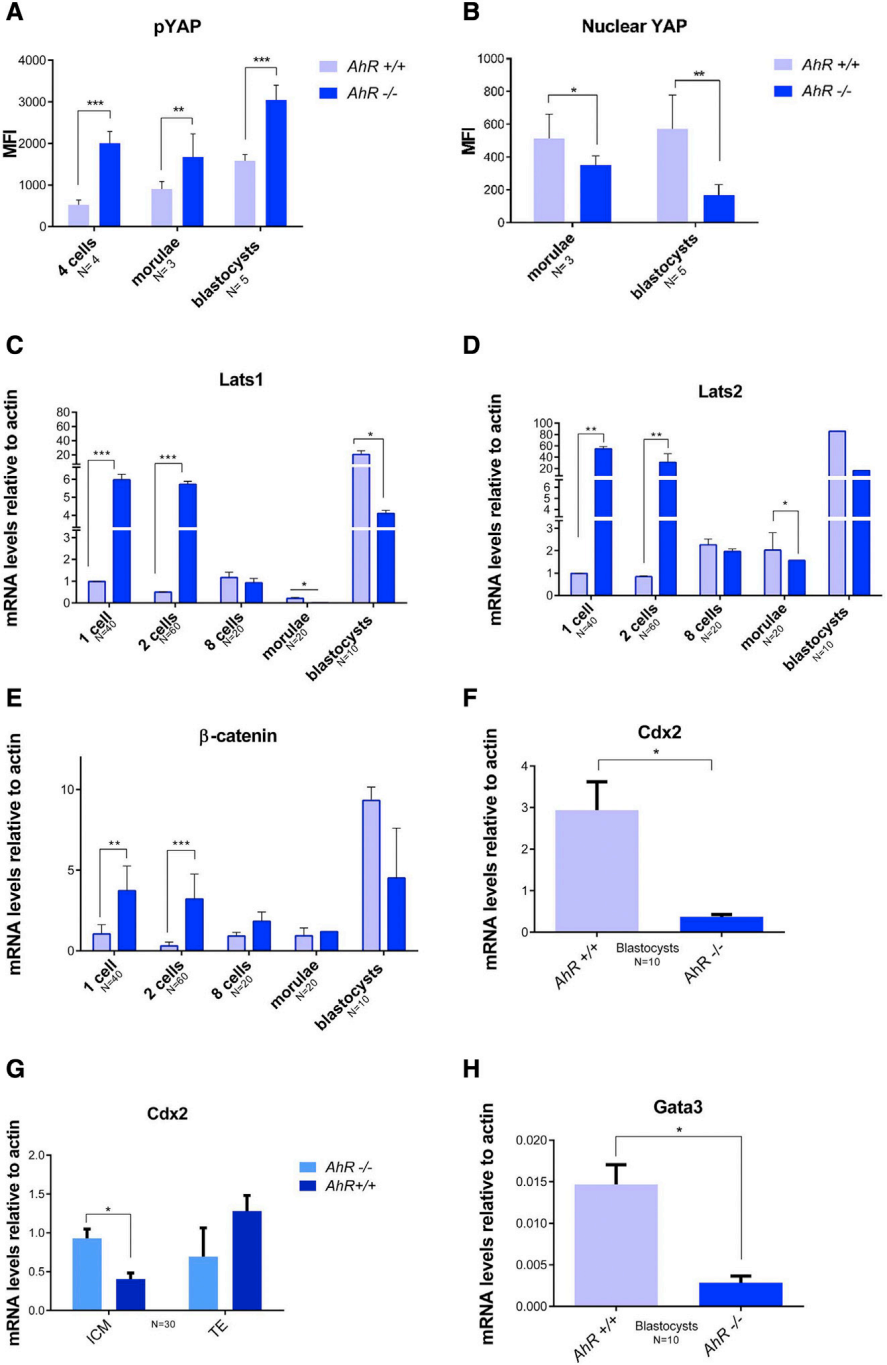
Hippo effectors and molecular intermediates of pluripotency are altered in the absence of AhR (A) Levels of pYAP were quantified from immunofluorescence and are presented as the mean fluorescence intensity (MFI). (B–H) Levels of nuclear YAP in morulae and blastocysts from *AhR*^+/+^ and *AhR*^−/−^ mice were quantified by immunofluorescence and are presented as the MFI (B). *AhR*^+/+^ and *AhR*^−/−^ embryos at the indicated developmental stages were used to purify embryo mRNA (C–F and H) or mRNA from TE and ICM fractions (G) that were separated by MACS as indicated in experimental procedures. The expression of *Lats1* (C), *Lats2* (D), *β-catenin* (E), *Cdx2* (F and G), and *Gata3* (H) was quantified by qRT-PCR. Expression levels were normalized by *β-actin* and are represented as 2^−ΔΔCt^. ^∗^p < 0.05; ^∗∗^p < 0.01; ^∗∗∗^p < 0.001. Data are shown as mean ± SD. The experiments were performed at least three times, and number of embryos analyzed is indicated on the x axis.

As indicated above, the Hippo pathway is implicated in cell polarity and cell fate. Next, we explored whether the effects of AhR on embryonic differentiation could be mediated through the Hippo pathway. To investigate such a possibility, we first analyzed the nuclear localization of the Hippo effector YAP. YAP was excluded from the cell nucleus in AhR-null embryos during most embryo development, whereas it had nuclear localization in external blastomeres in wild-type embryos from the morulae stage (Figure 5A). Immunofluorescence analysis indicated that pYAP was predominantly excluded from the cell nucleus in a fraction of blastomeres in AhR-null blastocysts (Figure 5B). Quantification of the mean fluorescence intensity (MFI) revealed that pYAP levels (e.g., cytosolic) were significantly higher in *AhR*^−/−^ embryos (Figure 6A) and, consequently, that the amounts of nuclear YAP (unphosphorylated) were reduced in the absence of AhR (Figure 6B). To further analyze the implication of the Hippo pathway, we measured the mRNA expression of the kinases responsible for YAP phosphorylation *Lats1* and *Lats2*. The results showed that their expression was significantly higher at the beginning of development (one-cell and two-cell) in embryos lacking *AhR*^−/−^ as compared with wild-type counterparts (Figures 6C and 6D). Interestingly, the levels of both kinases transiently decreased from eight-cell to morulae to increase again at the blastocyst stage (Figures 6C and 6D). In addition, β-catenin, a component of the complex located at adherent junctions where AMOT is retained, was overexpressed at the initial stages of development in *AhR*^−/−^ embryos (Figure 6E). Moreover, the transcriptional YAP target *Cdx2* was repressed in AhR-null blastocysts with respect to their wild-type counterparts (Figure 6F). In addition, *Cdx2* mRNA expression was higher in TE than in ICM of *AhR*^+/+^ blastocysts, whereas no significant differences were found in *Cdx2* distribution between the ICM and TE of *AhR*^−/−^ blastocysts (Figure 6G), further supporting increased activation of the Hippo pathway and reduced transcriptional activity of the OCT4 repressor YAP in the absence of AhR. Gata3, a trophectoderm marker in blastocysts, had lower mRNA levels in *AhR*^−/−^ embryos at the blastocyst phase (Figure 6H), supporting that lack of AhR promotes a more undifferentiated phenotype in preimplantation mouse embryos. Altogether, these data suggest that absence of AhR affects differentiation of the TE and ICM as the two first cell lineages established in the embryo.

### Embryos lacking AhR show a higher glycolytic metabolic activity and a lower rate of oxidative metabolism

**Figure 7:**
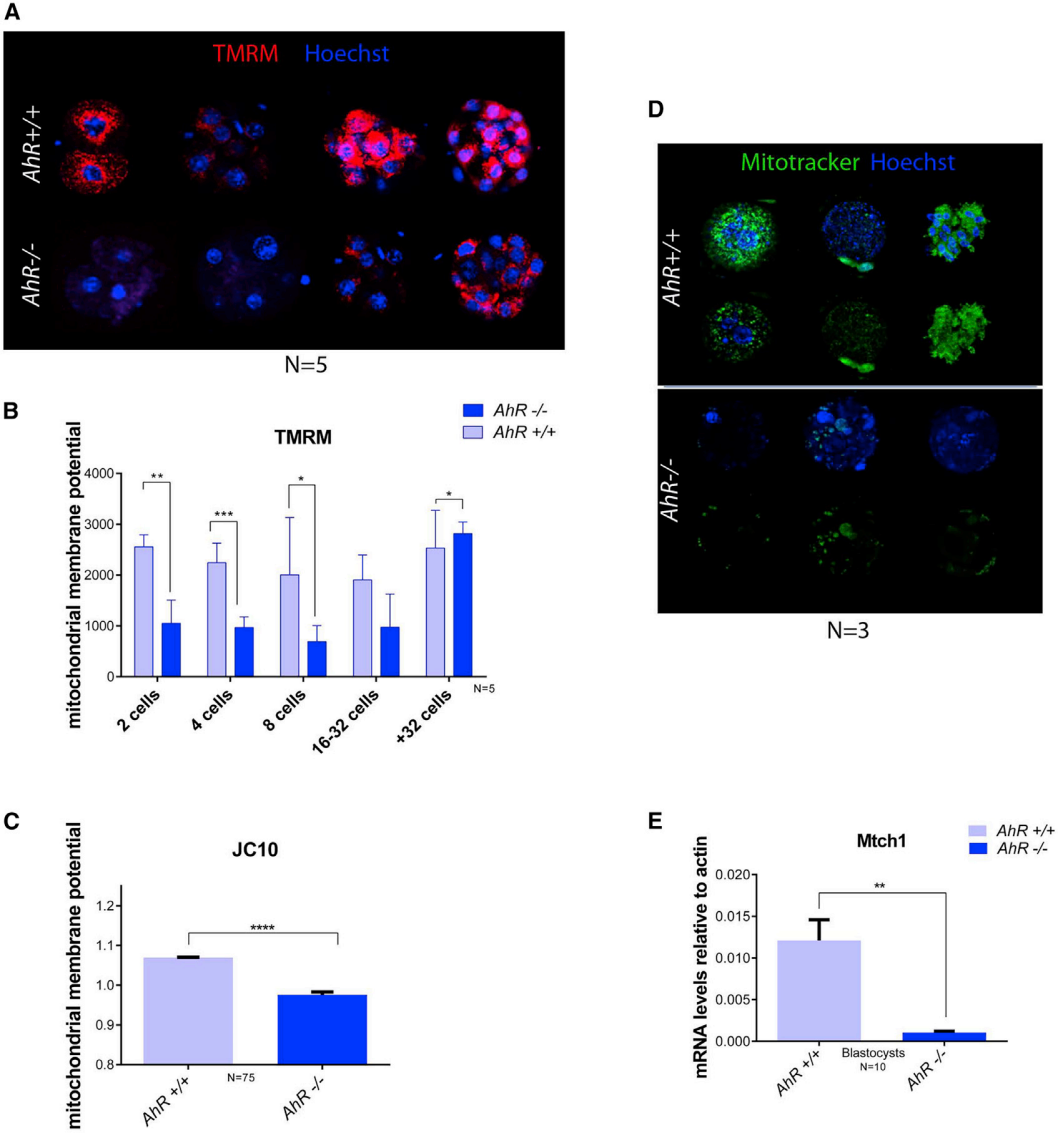
AhR-lacking embryos have lower mitochondrial activity (A and B) Mitochondrial membrane potential was measured by TMRM staining at the indicated developmental stages and then analyzed by confocal microscopy. Three embryos per genotype were analyzed. Scale bar, 20 μm. (C) Mitochondrial membrane potential was determined by JC10 staining in pools of 15–20 embryos. (D) Mitochondrial volume was analyzed by mitotracker green staining and visualized by confocal microscopy. Scale bar, 10 μm. (E) Mitochondrial carrier homolog-1 (*Mtch1*) mRNA expression was measured in blastocysts for each genotype and quantified by RT-qPCR. Expression levels were normalized by *β-Actin* and represented as 2^−ΔΔCt^. ∗p < 0.05; ∗∗p < 0.01; ∗∗∗p < 0.001. Data are shown as mean ± SD. The experiments were performed, at least, 3 times and number of embryos analyzed is indicated in the x axis.

The more undifferentiated status of *AhR*^−/−^ embryos could be associated with a more immature physiological phenotype. We next decided to study glycolytic and oxidative metabolism rates, since it is well established that these two parameters are strongly linked to the pluripotency state of embryonic stem cells. First, we analyzed the mitochondrial membrane potential of embryos of both genotypes at different stages using tetramethyl rhodamine (TMRM) staining. Embryos lacking AhR maintained a lower mitochondrial activity until the 32-cell stage, while wild-type embryos had a significantly higher mitochondrial activity during the same period (Figures 7A and 7B). To further assess this result, we collected pools of *AhR*^−/−^ and *AhR*^+/+^ embryos and analyzed their mitochondrial membrane potential using the JC-10 probe. The results confirmed that the mitochondrial membrane potential was higher in wild-type than in AhR-null embryos (Figure 7C). Moreover, the mitochondrial volume measured by MitoTracker green staining was significantly lower in *AhR*^−/−^ than in *AhR*^+/+^ embryos (Figure 7D), as well as the mRNA levels of the marker for mitochondrial activity mitochondrial carrier homolog-1 (*Mtch1*) (Figure 7E). These results indicate that lack of AhR may contribute to a lower rate of oxidative metabolism in the mouse embryo.

Next, we investigated whether glycolytic metabolism, the preferred energy source for pluripotent and cancerous cells, would be influenced by AhR activity throughout embryonic differentiation. We observed that the mRNA expression of the hexokinase enzyme (*HK*) and of the glucose transporters *Scl2a1* and *Scl2a3* were increased in *AhR*^−/−^ compared with *AhR*^+/+^ blastocysts (Figures S2A–S2C). We then decided to measure hexokinase activity using an enzymatic assay in embryos at developmental stages between morulae and blastocyst. The results obtained showed that absence of AhR generated a significant increase in hexokinase activity (Figure S2D). The less differentiated status of AhR-deficient embryos with respect to wild-type ones correlates with their preferential glycolytic metabolism. Thus, lack of AhR alters mitochondrial functions that are consistent with a more pluripotent phenotype. Stem cells specifically use the amino acid threonine to maintain their pluripotent status, and such a cellular condition is dependent on the activity of threonine dehydrogenase (TDH) (Rey-Barroso et al., 2013). We have found that *Tdh* mRNA expression was increased in the absence of AhR along embryonic development from one-cell zygote to blastocyst (Figure S2E), supporting that AhR-null preimplantation embryos have an altered metabolic profile.

## Discussion

AhR promotes cell differentiation through the inhibition of pluripotency genes. As a result, from AhR deficiency an undifferentiated phenotype originates not only in cell lines but also in models of tissue regeneration in mice (Laiosa et al., 2015; Roman et al., 2018; Wang et al., 2016). However, our knowledge about the role of AhR in embryo differentiation is still very limited, in particular with respect to the molecular intermediates that might be regulated by AhR. This encouraged us to investigate the role of AhR in embryonic stem cell differentiation *in vivo* during mouse preimplantation embryonic development. In this phase of embryogenesis, totipotent blastomeres generate the first three cell lineages of the embryo: trophectoderm, epiblast, and PE. Mouse embryogenesis has been widely studied to aid in understanding developmental processes in mammals, but also constitutes an excellent model for studying the plasticity of stem cells. Understanding how molecular intermediates govern the balance between pluripotency and differentiation in blastocyst development allows us to understand stem cell behavior in other physiological and pathological conditions. An important finding of this study is that AhR affects the differentiation processes of embryo development by interacting with different signaling networks.

As cleavage of the early zygote takes place, central pluripotency factors increase their expression to produce totipotent cells that will proliferate and differentiate to generate a complete organism. We have first found that zygotes from *AhR*^−/−^ mice have basal overexpression of well-known pluripotency factors *Oct4*, *Nanog*, and *Sox2*, in agreement with our previous studies reporting that AhR-null mice have an increased ability to regenerate lung (Laiosa et al., 2015) and liver (Wang et al., 2016), and a higher potential to sustain undifferentiation of human embryonic carcinoma cells (Roman et al., 2018). The apparent global role of AhR in controlling differentiation was also reported by its ability to regulate ovarian follicular development through Piwi-interacting RNA (piRNA)-associated proteins, piRNAs, and retrotransposons (Ozawa and Hansen, 2011).

Pluripotency genes need to reach a certain expression level to activate the networks that control pluripotency. Interestingly, *Oct4*, *Nanog*, and *Sox2* reached their highest expression levels in a transient manner in one-cell and two-cell *AhR*^−/−^ embryos, suggesting that their atypical upregulation very early in development could affect proper embryo differentiation and contribute to the deficient ability of *AhR*^−/−^ mice to sustain implantation and *in utero* survival (Bedzhov et al., 2014; Boyer et al., 2006; Wang et al., 2009). The pro-differentiation role of AhR in embryogenesis is also supported by its own regulation during the process. AhR levels increased with differentiation and, interestingly, its location was mainly restricted to the blastomeres that differentiate to form the trophectoderm, indicating that AhR may exert a differential regulatory function limiting pluripotency in those blastomeres that will generate extraembryonic tissues. In this sense, a central regulator such as OCT4 showed an expression pattern opposite to that of AhR, again supporting its repressive role in pluripotency. The crosstalk between OCT4 and AhR has been also suggested from studies using stem-like cancer cells that, in fact, proposed a reciprocal suppression between AhR and such a pluripotency factor (Fernandez-Salguero et al., 1995; Rico-Leo et al., 2016).

In the morulae, it is known that the first asymmetric division is determinant for embryonic differentiation and that, in the formed blastocyst, a second differentiating wave gives rise to two types of cells in the ICM. The fact that AhR expression was modulated during these processes, together with previous studies that link embryo differentiation to the Hippo pathway, lead us to think that AhR could act through Hippo in the phenotype observed. Our preliminary data indicate that nuclear YAP levels can be modulated by AhR to produce a more differentiated status in NTERA-2 cells. In this work, we have shown that the fate of TE cells seems to be activated by nuclear YAP in an AhR-dependent manner and thus that AhR and YAP co-localized in the nucleus of external blastomeres, eventually differentiating to the trophectoderm lineage. The fact that AhR-null blastocysts had OCT4 expression but lacked nuclear YAP in external blastomeres suggests that AhR deficiency may result in a failure to link polarity to transcription factors that lead to differentiation through Hippo signaling.

One characteristic of pluripotent cells is their low levels of oxidative phosphorylation and their preferred glycolytic ATP synthesis. Upregulation of glycolysis precedes the reactivation of pluripotent markers (Cheng et al., 2015). The differences that we have observed in differentiation markers through embryo development were correlated with their metabolic status. Lack of AhR reduced mitochondrial activity and maintained a predominant glycolytic metabolism. During differentiation, metabolic pathways are modulated according to the energetic needs of the embryo. Our results are in agreement with these hypotheses, since *AhR*^−/−^ embryos overexpressed TDH, which is an enzyme responsible for providing metabolites generated from Thr specifically used for stem cell self-renewal. Therefore, lack of AhR probably causes a metabolic state in the embryos that corresponds to a lower differentiation state. Previous studies have shown that the transcription factor TEAD4 plays a crucial role in maintaining energy homeostasis during preimplantation development (Kaneko and DePamphilis, 2013). Also, the transcriptional output of the Hippo pathway is mediated by TEAD proteins that partner with YAP to activate genes that stimulate cell proliferation (Hansen et al., 2015; Marti et al., 2015). The link of Hippo signaling to the mitochondrial activity and the interaction of YAP1 with TEAD4 transcription factor opens new insights to be explored.

In summary, AhR has relevant functions in embryonic development, adjusting the expression of signaling pathways that control pluripotency and differentiation. Under low AhR levels, a defective differentiation status may compromise completion of the embryo developmental program, implantation, and survival.

## Experimental procedures

### Embryo collection

C57BL/6N wild-type (AhR^+/+^) and AhR-null (AhR^−/−^) mice were kept under a 12-h light/dark cycle and had free access to rodent chow and water. Four- to 7-week-old females were injected with 7.5 IU of pregnant mare's serum gonadotropin followed 48 h later by intraperitoneal injection of 5 IU of human chorionic gonadotropin (hCG). Females were sacrificed at the indicated developmental stages, and the oviducts/hemiuterus were collected in PBS and flashed for embryo collection. Embryos were isolated using a stripper (Origio). Embryos were considered to be one-cell zygote at 1.5 days after hCG induction. Blastocysts (late) were isolated at 3.5 days following hCG administration. The number of cells during morulae is indicated in the corresponding figures.

All work involving mice was performed in accordance with the National and European legislation (Spanish Royal Decree RD53/2013 and EU Directive 86/609/CEE as modified by 2003/65/CE, respectively) for the protection of animals used for research. Experimental protocols using mice were approved by the Bioethics Committee for Animal Experimentation of the University of Extremadura (Registry 109/2014) and by the Junta de Extremadura (EXP-20160506-1).

### Gene expression analysis

Total RNA was isolated from mouse embryos using the Pico Pure RNA isolation kit (Thermo Fisher) and purified following the manufacturer's instructions. Reverse transcription was performed using random priming and iScript Reverse Transcription Super Mix (Bio-Rad). Real-time PCR was used to quantify the mRNA expression of AhR, Nanog, Oct4, Sox2, Lats1, Lats2, β-catenin, Scl2a1, Scl2a2 (Solute carrier family 1 and 2), hexokinase, Cdx2, Gata3, and TDH (L-threonine 3-dehydrogenase). Reactions were carried out using Luna Master Mix (New England Biolabs) in a Step One thermal cycler (Applied Biosystems) essentially as described by Abbott et al. (1999). The expression of β-actin was used to normalize gene expression (ΔCt), and 2^−ΔΔCt^ was applied to calculate changes in RNA levels with respect to control conditions. Primer sequences used are indicated in Table S1.

### Whole-mount immunofluorescence

Each group of embryos was independently fixed in 3.5% paraformaldehyde for 15 min at room temperature. The zona pellucida was removed by incubation in Tyrode's acid solution for 15–20 s at 37°C. Embryos were blocked in PBS containing 1% BSA and 0.1 M glycine for 2.5 h followed by incubation in blocking solution with antibodies against NANOG (Novus Biologicals, NBP2-13177) diluted 1:150, OCT4 (Santa Cruz Biotechnology, Sc-5279) diluted 1:75, AhR (BML-SA210-0100) diluted 1:50, YAP (NB110-58358) diluted 1:150, and pYAP (PA5-17481) diluted 1:75 overnight at 4°C. Following washings, secondary antibodies labeled with Alexa 647 (Invitrogen, A32728 and A322733), Alexa 488 (Invitrogen, A32723 and A32731), or Alexa 555 (Invitrogen, A32727 and A32732) were added for 2 h at 4°C at 1:350 dilution. Samples were further washed and incubated with Hoechst to stain cell nuclei. Embryos were transferred to ibidi chambers and analyzed using an Olympus FV1000 confocal microscope. Fluorescence analysis was done using the FV10 software (Olympus) and ImageJ software.

### Magnetic-activated cell sorting

ICM and TE cells were separated using concanavalin and MACS microbeads essentially as described by Ozawa and Hansen (2011). At 96 h after hCG injection, blastocysts were harvested and incubated in acidic Tyrode's solution to remove the zona pellucida. Samples were washed three times in MACS buffer (Dulbecco’s PBS with 0.5% [w/v] BSA and 2 mM EDTA [pH 7.2]) and incubated for 10 min with concanavalin A conjugated fluorescein isothiocyanate (ConA-FITC, Sigma-Aldrich; 1 mg/mL in MACS buffer). Following three washes in MACS buffer, blastocysts were incubated in PBS containing 1 mM EDTA for 5 min followed by incubation in 0.05% (w/v) trypsin/0.53 mM EDTA solution (Invitrogen) for 10 min at 37°C. Groups of 15–20 blastocysts were disaggregated into single blastomeres by pipetting with a stripper (Origen) under a dissecting microscope. Blastomeres were then transferred to PBS containing 1 mM EDTA and 10% (v/v) fetal bovine serum to stop the reaction. Next, samples were washed in MACS buffer by centrifugation at 500 × *g* for 5 min and resuspended in 110 μL of MACS buffer. Disaggregated blastomeres were then further incubated with 10 μL of magnetic microbeads conjugated to mouse anti-FITC (Miltenyi Biotec) for 15 min on ice. Following two washes by centrifugation at 500 × *g* for 5 min, samples were resuspended in 500 μL of MACS buffer and passed through MACS separation columns (Miltenyi Biotec) attached to a magnetic board (Spherotech). The FITC-negative fraction (ICM) was eluted by three 500-μL MACS buffer washes followed by FITC-positive elution (TE) by removing the MACS separation column from the magnetic board and washing three times with 500 μL of MACS buffer.

### TMRM and MitoTracker staining

Embryos were arranged in staining solution made by mixing 10 μL of 100 μM tetramethylrhodamine solution (Invitrogen) with 10 mL of KSOM medium (EmbrioMax; Millipore) or with 100 nM of MitoTracker green (Cell Signaling Technology). Embryos were placed on ibidi plates in a 5% CO_2_ incubator at 37°C for 30 min for TMRM staining and for an additional 20 min for MitoTracker. Embryos were then washed twice in PBS and analyzed by confocal microscopy.

### Mitochondrial potential measurement using the JC-10 kit

Pools of 25 embryos were placed in a 96-well plate and processed following the non-adherent cell protocol recommended by the manufacturer. An aliquot of 50 μL of JC-10 dye was added per well, and the embryos were incubated in a 5% CO_2_ incubator at 37°C for 30 min. Next, 50 μL of assay buffer was added and fluorescence intensity was monitored in a fluorescence multiwell plate reader using excitation wavelength of 490 nm and emission wavelength of 525 nm. For ratio analysis, signals were also recorded at excitation wavelength of 540 nm and emission wavelength of 590 nm. The red/green fluorescence intensity ratio was then used to determine the mitochondrial membrane potential.

### Hexokinase activity measurement

Groups of 20 blastocysts were disaggregated by incubation in 0.05% (w/v) trypsin solution containing 0.53 mM EDTA for 10 min at 37°C. Samples were centrifuged, washed twice in PBS containing 1 mM EDTA and 10% (v/v) fetal bovine serum, and washed once in PBS. Single blastomeres were resuspended in hexokinase assay buffer and homogenized through passage by a 30-gauge syringe. Homogenized samples were used in the Pico Probe hexokinase activity assay kit (Biovision) following the manufacturer's indications.

### Statistical analyses

GraphPad Prism 6.0 software (GraphPad) was used to perform comparison between experimental conditions. Student's t test (unpaired two-sided) was used to analyze differences between two experimental groups. For three or more experimental conditions data were analyzed using ANOVA. Data are shown as mean ± SD. Differences were considered significant at ^∗^p<0.05, ^∗∗^p<0.01, and ^∗∗∗^p<0.001. Data analyses are indicated in the figure legends.

## Author contributions

P.M.F.-S. conceived the study. A.N.-P., F.J.G.-R., C.R.-G., and A.O.-T. performed the experiments. P.M.F.-S. and J.M.M. supervised data analysis. P.M.F.-S. wrote the manuscript with input from all other authors.

## Declaration of interests

The authors declare no competing interests.

## Supplemental information

Document S1. Figures S1 and S2 and Table S1

Document S2. Article plus supplemental information
