# Supplementary material for: The aryl hydrocarbon receptor promotes differentiation during mouse preimplantational embryo development
Source: Stem Cell Reports. 2021 Sep 2;16(9):2351–63. doi: 10.1016/j.stemcr.2021.08.002 (PMC8452532; doi:10.1016/j.stemcr.2021.08.002)
Supplement: Document S1. Figures S1 and S2 and Table S1 [file mmc1.pdf]

Stem Cell Reports, Volume *16*

Supplemental Information

The aryl hydrocarbon receptor promotes differentiation during mouse preimplantational embryo development

Ana Nacarino-Palma, Francisco J. González-Rico, Claudia M. Rejano-Gordillo, Ana Ordiales-Talavero, Jaime M. Merino, and Pedro M. Fernández-Salguero

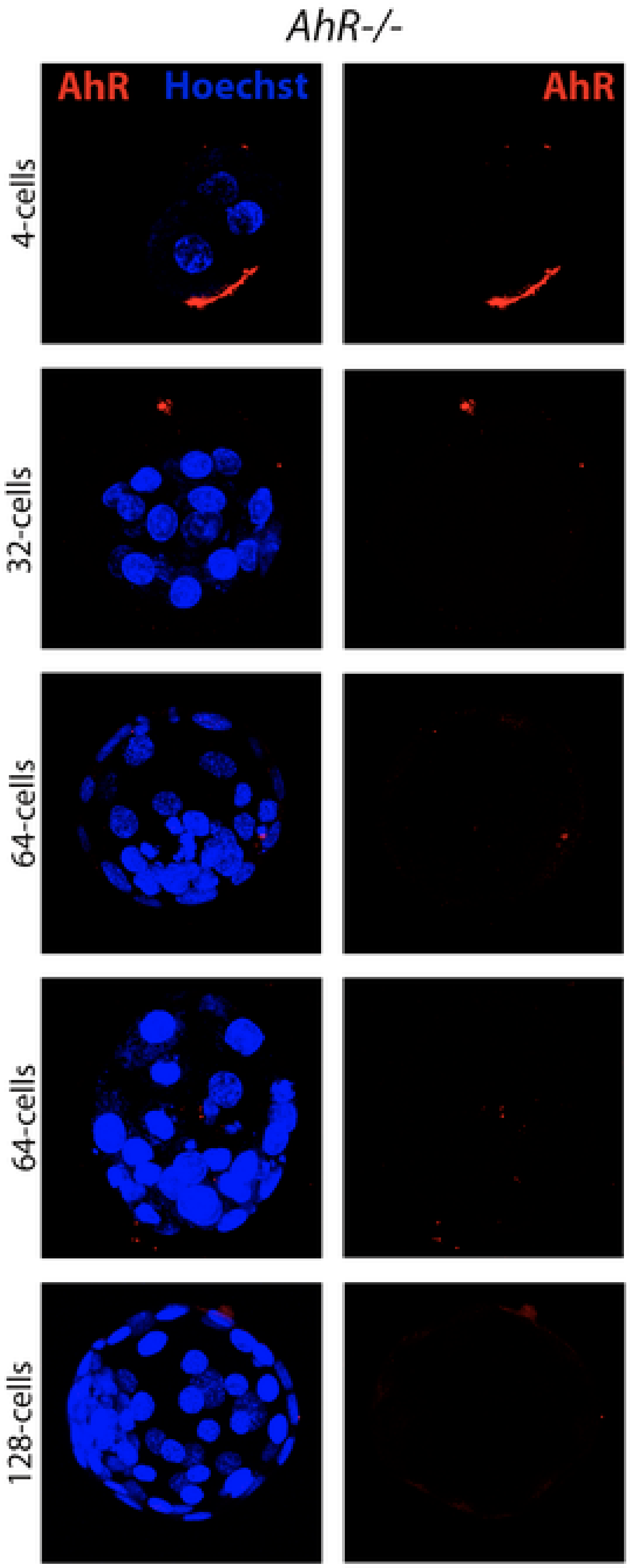

Supplementary Figure 1. AhR expression was determined in 4-cells, 32-cells, 64-cells and 128-cells AhR-null mouse embryos by immunofluorescence. Hoechst staining was used to label cell nuclei. Confocal microscopy was used for detection using an Olympus FV1000 confocal microscope.

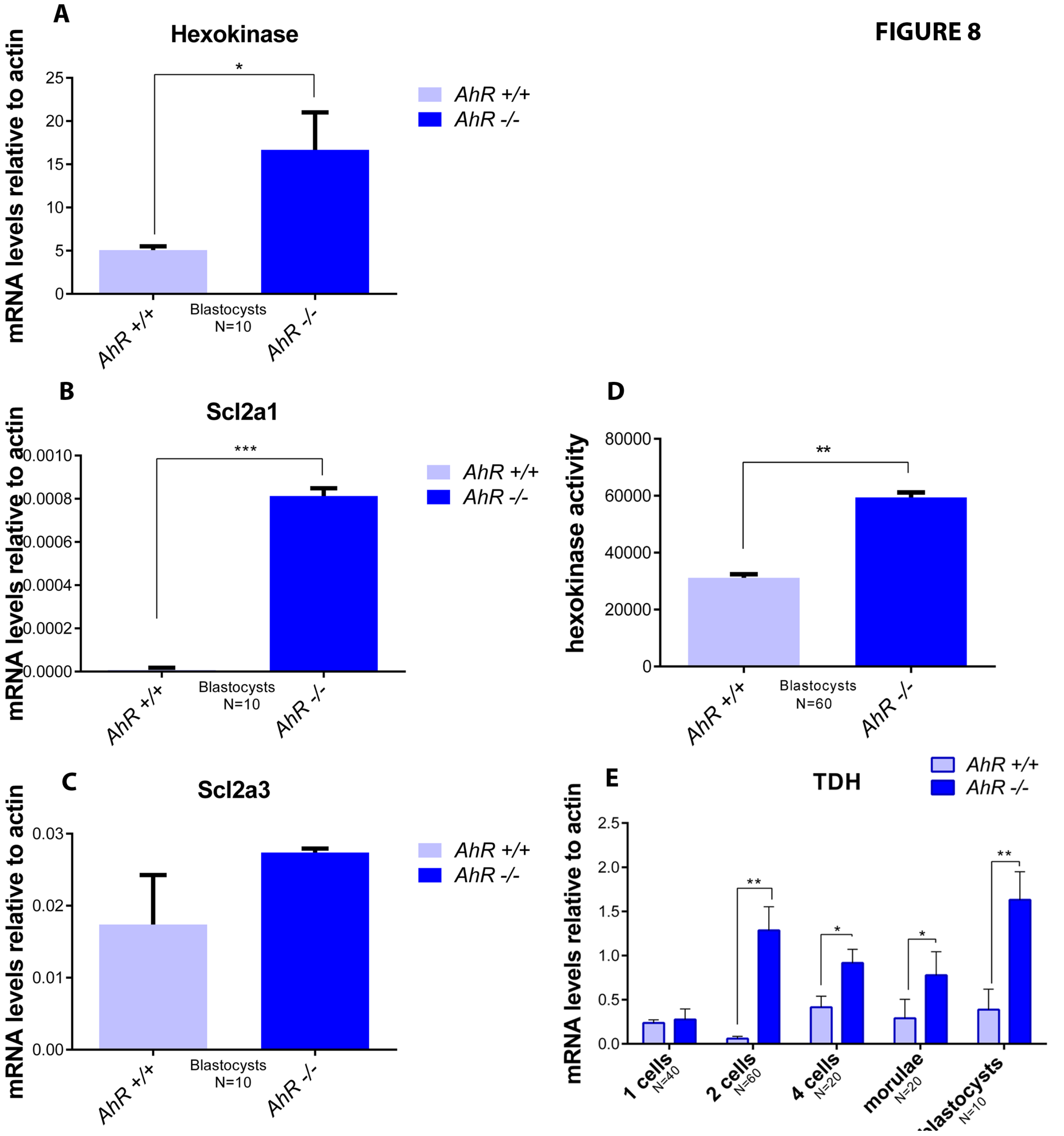

Supplementary Figure 2. AhR depletion favors a glycolytic metabolism. mRNA was purified from *AhR+/+* and *AhR-/-* blastocysts and the expression of *Hexokinase-HK* **(A)**, Scl2a1 **(B)** and *Scl2a3* **(C)** was quantified by RT-qPCR. Expression levels were normalized by *β-Actin* and represented as $2^{-\Delta\Delta Ct}$. **(D)** Hexokinase activity was measured using pools of 25-30 embryos at the morulae to blastocyst stages by an enzymatic assay. **(E)** Threonine dehydrogenase expression was analyzed at the indicated developmental stages in *AhR+/+* and *AhR-/-* embryos. *p< 0.05; **p< 0.01; ***p< 0.001. Data are shown as mean ± SD. The experiments were performed, at least, 4 times (2 times for D panel) and the number of embryos analyzed is indicated in the x axis.

Supplementary Table S1

Oligonucleotide sequences used for mRNA expression analysis

Gene	Sequence 5´-- 3´
AhR	Fw: ACATCACCTACGCCAGTCGC<br>Rv: TCTATGCCGCTTGGAAGGAT
Nanog	Fw: CAAGGGTCTGCTACTGAGATGCTCTG<br>Rv: TTTTGTTTGGGACTGGTAGAAGAATCAG
Oct4	Fw: AGAGGGAACCTCCTCTGAGC<br>Rv: CCAAGGTGATCCTCTTCTGC
Sox2	Fw: CGTAAGATGGCCCAGGAGAA<br>Rv: GCTTCTCGGTCTCGGACAAA
β-catenin	Fw: GTGCAATTCCTGAGCTGACA<br>Rv: CTTAAAGATGGCCAGCAAGC
Lats1	Fw: GACCCCAGCTAATGGACAAA<br>Rv: GTTTGCAGTCCAGGGACATT
Lats2	Fw: GTGTCCACAAGATGGGCTTT<br>Rv: CTCCATGCTGTCCTGTCTCA
Cdx2	Fw: TCTCCGAGAGGCAGGTTAAA<br>Rv: GCAAGGAGGTCACAGGACTC
Gata3	Fw: CCGAAACCGGAAGATGTC<br>Rv: TCAGCATGTGGGTGGAGT
Mtch1	Fw: CAGAATCCAGGTTCCCAGTT<br>Rv: TCAGGTACTTCCAGCAGTGG
Scl2a1	Fw: CCCCAGAAGGTTATTGAGGA<br>Rv: GGTTCATCATCAGCATGGAG
Scl2a3	Fw: CTTATGGGATTCGCCAAGAT<br>Rv: TACCAGAATCCCAACAACGA
Tdh	Fw: AAGCTTGTCTTGCCCTTGAA<br>Rv: CTTCCCAAATCGTTTCCTCA
Hexokinase	Fw: TGTTCGAGAAGATGGTGAGC<br>Rv: CATACGTGCTGGACCGATAC
